# Recombinant BA.1/BA.2 SARS-CoV-2 Virus in Arriving Travelers, Hong Kong, February 2022

**DOI:** 10.3201/eid2806.220523

**Published:** 2022-06

**Authors:** Haogao Gu, Daisy Y.M. Ng, Gigi Y.Z. Liu, Samuel S.M. Cheng, Pavithra Krishnan, Lydia D.J. Chang, Sammi S.Y. Cheuk, Mani M.Y. Hui, Tommy T.Y. Lam, Malik Peiris, Leo L.M. Poon

**Affiliations:** The University of Hong Kong, Hong Kong, China (H. Gu, D.Y.M. Ng, G.Y.Z. Liu, S.S.M. Cheng, P. Krishnan, L.D.J. Chang, S.S.Y. Cheuk, M.M.Y. Hui, T.T.Y. Lam, M. Peiris, L.L.M. Poon);; Centre for Immunology & Infection, Hong Kong (T.T.Y. Lam, M. Peiris, L.L.M. Poon);; HKU-Pasteur Research Pole, Hong Kong (M. Peiris, L.L.M. Poon)

**Keywords:** COVID-19, respiratory infections, severe acute respiratory syndrome coronavirus 2, SARS-CoV-2, SARS, coronavirus disease, zoonoses, viruses, coronavirus, Hong Kong

## Abstract

We studied SARS-CoV-2 genomes from travelers arriving in Hong Kong during November 2021–February 2022. In addition to Omicron and Delta variants, we detected a BA.1/BA.2 recombinant with a breakpoint near the 5′ end of the spike gene in 2 epidemiologically linked case-patients. Continued surveillance for SARS-CoV-2 recombinants is needed.

The SARS-CoV-2 Omicron variant (Pango lineage B.1.1.529) emerged in November 2021. Within a few weeks, subvariants BA.1, BA.1.1, and BA.2 were detected in varying proportions on different continents, but BA.1 initially was dominant (*1*). By March 2022, these 3 subvariants accounted for >95% of sequences submitted to GISAID (https://www.gisaid.org). We previously demonstrated the feasibility of testing incoming travelers for SARS-CoV-2 genomic surveillance (*2*). We report detecting a BA.1/BA.2 recombinant SARS-CoV-2 subvariant in travelers arriving in Hong Kong, China.

Using our previously described next-generation sequencing method (*2*), we analyzed 198 (25%) of 793 SARS-CoV-2 reverse transcription PCR (RT-PCR)–positive samples collected from travelers arriving in Hong Kong during November 15, 2021–February 4, 2022 (Appendix Table 1). We randomly selected samples with cycle thresholds <30 and successfully deduced near-full genome sequences from 180 samples (mean coverage 97.6%; depth >100). Deduced genomes predominantly were Delta (n = 58) and Omicron (BA.1 = 66, BA.1.1 = 28, and BA.2 = 26) variants (Appendix Figures 1, 2). Time distribution of these variants agrees with global surveillance data submitted to GISAID, confirming that travel hubs are useful sentinel sites to monitor SARS-CoV-2 circulation (*2*). Of note, the BA.2 cases we detected predominantly were imported from the Philippines and Nepal, indicating this subvariant might have become established in these countries before detection in Hong Kong.

In our phylogenetic analysis, 2 additional nearly identical sequences formed a distinct branch in the Omicron clade (Appendix Figure 2). We detected these sequences from 2 epidemiologically linked cases, patients 1 and 2, who were work colleagues and traveled together to Hong Kong on February 1, 2022, from Germany via the Netherlands. They tested SARS-CoV-2–positive by RT-PCR at the airport upon arrival (cycle thresholds 27 and 22). Patient 1 reported having a sore throat and cough since January 28, but patient 2 was asymptomatic. Both patients had received 2 doses of Pfizer-BioNTech COVID-19 vaccine (Pfizer Inc., https://www.pfizer.com); patient 1 received the second dose on November 1, 2021, and patient 2 received the second dose on June 22, 2021.

The distinct topology of viral sequences from these patients suggested that they were infected by a recombinant virus. To test that hypothesis, we used previously reported BA.1- and BA.2-defining single-nucleotide polymorphisms (SNPs) to analyze the genomes (Appendix). We found that the 5′ end sequences (nucleotide region 1–20055) from the 2 cases only contained BA.1-specific SNPs (Figure, panel A). By contrast, the corresponding 3′ end sequences only contained BA.2-specific SNPs. We further conducted a recombination analysis and confirmed that only 1 breakpoint was located within nucleotide positions 20055–21618 (Appendix Figure 3). The nucleotide 5′ end of the sequences is phylogenetically similar to authentic BA.1 and the 3′ end similar to BA.2 sequences at this breakpoint (Figure, panel B). The breakpoint identified in this recombinant virus is near the 5′ end open reading frame of the spike gene. Recombinant viruses, including B.1.1.7/B1.177 and Delta/BA.1, with a breakpoint in this region have been reported (*3*; P. Colson et al., unpub. data, https://www.medrxiv.org/content/10.1101/2022.03.03.22271812v2; T. Peacock, unpub. data, https://github.com/cov-lineages/pango-designation/issues/441).

**Figure:**
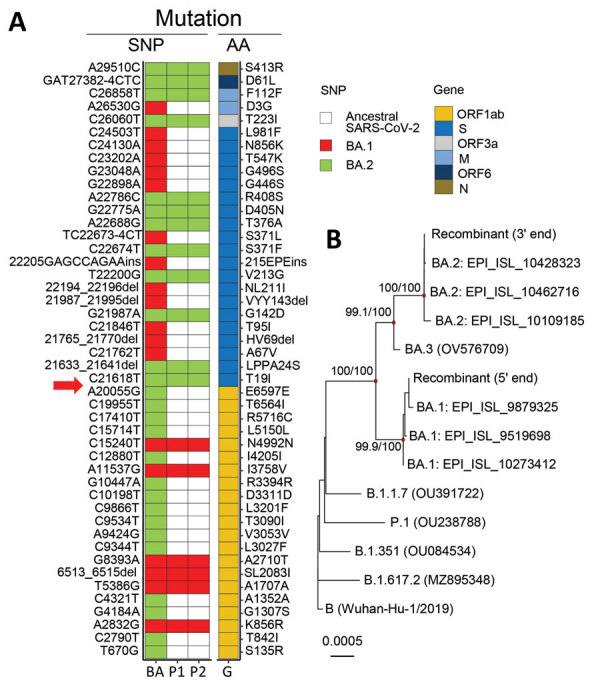
Detection of recombinant BA.1/BA.2 SARS-CoV-2 virus in arriving travelers, Hong Kong, China, February 2022. A) Mapping of BA.1- and BA.2-specific SNPs against the reference sequence genome (Genbank accession no. MN908947.3). Red boxes indicate BA.1-specific SNPs and green boxes indicate BA.2-specific SNPs found in samples from P1 and P2; the corresponding AA changes of these SNPs also are indicated. Red arrow indicates the putative breaking point. B) Phylogeny of viral RNA sequences at the 5′ and 3′ ends to the putative breakpoint. The maximum-likelihood tree was generated by using IQ-TREE (http://www.iqtree.org) and the transition plus empirical base frequencies plus proportion of invariable site nucleotide substitution model with Wuhan-Hu-1 (GenBank accession no. MN908947.3) as the outgroup. References sequences are shown with GISAID (https://www.gisaid.org) or GenBank accession numbers. Red node points show strongly supported branches as detected by SH-aLRT and ultrafast bootstrap values. Scale bar indicates nucleotide substitutions per site. AA, amino acid; BA, BA.1/BA.2 recombinant; G, gene; M, membrane; N, nucleocapsid; ORF, open reading frame; P1, patient 1; P2, patient 2; S, spike; SNP, single-nucleotide polymorphism.

We further examined our sequence data to exclude the possibility of coinfection or contamination (*4*). We noted that the minor allele frequencies at these BA.1- and BA.2-defining SNP positions were extremely low (mean 0.5%, median 0.06%) (Figure, panel A), indicating these samples contained only 1 virus population. We used the patient 2 sample to clone an RT-PCR product (≈2.2 kbp) spanning the recombination breakpoint. We detected BA.1-specific (19955C/20055A) and BA.2-specific (21618T/21633–21641del/21762C) SNPs in the same plasmid clone, confirming the 2 patients were infected by a BA.1/BA.2 recombinant virus.

We found no similar BA.1/BA.2 recombinant sequences in GISIAD or GenBank (as of March 7, 2022), suggesting a novel recombinant. The BA.1 region of this recombinant virus is genetically close to 3 BA.1 sequences detected in Europe and the United States (Figure, panel B), whereas its BA.2 region is identical to 19,555 BA.2 sequences from multiple continents. Because global cocirculation of BA.1 and BA.2 subvariants is high, pinpointing the geographic location where this recombination event occurred would be difficult.

Emerging Omicron subvariants could allow vaccine breakthrough and widespread reinfection. Previous studies reported detection of SARS-CoV-2 interlineage recombinants at the same time as different SARS-CoV-2 lineages were cocirculating (*3*; D. VanInsberghe et al., unpub. data, https://doi.org/10.1101/2020.08.05.238386; P. Colson et al., unpub. data; T. Peacock, unpub. data). The high transmissibility of Omicron (*5*,*6*) has led to wide cocirculation of BA.1 and BA.2 subvariants in many regions, which might provide ample opportunities to generate novel recombinants among these or other variants via coinfection events. Although current global surveillance data suggest that our recombinant might only be a sporadic case, the potential effects of novel recombinants should not be underestimated. Of note, homologous recombination is common in animal and other human coronaviruses (*7*), and some recombination events could generate recombinants with enhanced virulence (*8*,*9*). Long-term global SARS-CoV-2 genomic surveillance will be needed to monitor for possible more virulent or transmissible strains.

Appendix Additional information recombinant BA.1/BA.2 SARS-CoV-2 virus in arriving travelers, Hong Kong, China, February 2022.
